# Trait anxiety reduces affective fading for both positive and negative autobiographical memories

**DOI:** 10.5709/acp-0159-0

**Published:** 2014-09-30

**Authors:** W. Richard Walker, Cecile N. Yancu, John J. Skowronski

**Affiliations:** 1 Department of Psychological Sciences, Winston-Salem State University, USA; 2 Department of Behavioral Sciences, Winston-Salem State University, USA; 3 Department of Psychology, Northern Illinois University, USA

**Keywords:** trait anxiety, fading affect bias, emotion, autobiographical memory

## Abstract

The affect associated with negative events fades faster than the affect associated with positive events (the Fading Affect Bias; the FAB). The research that we report examined the relation between trait anxiety and the FAB. Study 1 assessed anxiety using the Depression, Anxiety, and Stress Scale; Studies 2 and 3 used the Beck Anxiety Inventory. Studies 1 and 2 used retrospective procedures to probe positive event memories and negative event memories while Study 3 used a diary procedure. The results of all 3 studies showed that increased anxiety was associated with both a lowered FAB and lower overall affect fading for both positive events and negative events. These results suggest that for people free of trait anxiety, the FAB reflects the operation of a healthy coping mechanism in autobiographical memory that is disrupted by trait anxiety.

## Introduction

Research suggests that the affect associated with most autobiographical events fades over time. The fading, however, is generally greater for the affect associated with negative events than for the affect associated with positive events. This pattern of differential affective fading has been termed the *Fading Affect Bias* (FAB; Walker, Skowronski, & Thompson, 2003) and has been the target of considerable systematic study (Ritchie, Skowronski, Wood, Walker, Vogl, & Gibbons, 2006; Skowronski, Gibbons, Vogl, & Walker, 2004; Walker, Skowronski, Gibbons, Vogl, & Thompson, 2003; Walker & Skowronski, 2009; Walker, Vogl, & Thompson, 1997).

The FAB has been documented using a host of different methods (for a review, see Skowronski, Walker, Henderson, & Bond, 2014). For example, Landau and Gunter (2009) showed that the FAB occurs regardless of whether event types are collected within-subject or between-subjects and irrespective of the order in which ratings of event emotions are obtained. Similarly, Ritchie, Skowronski, Hartnett, Wells, and Walker (2009) showed that the FAB effect occurred regardless of whether emotions assessed were active (elated, angry) or passive (calm, sad): Activation level, albeit related to the overall fading of affect, was unrelated to the emergence of the FAB.

However, the emergence of the FAB does depend on both the events recalled and the characteristics of those who recall the events. For example, Ritchie et al. (2006) found that the magnitude of the FAB was especially small when autobiographical events were self-important, psychologically open (Beike & Wirth-Beaumont, 2005), or self-caused. These results also showed that the FAB was especially large when events were considered to be atypical of a person’s life, when they were frequently rehearsed, and when they were rehearsed by means of discussing the events with others.

Individual differences also moderate the FAB. Walker, Skowronski, Gibbons, et al. (2003) examined the relation between non-clinical depression (dysphoria) and the FAB by assessing over 300 participants using the Beck Depression Inventory-II (BDI; see Beck, Steer, & Brown, 1996). A participant’s BDI score was used to place the participant into one of five groups. Each of these participants was also asked to retrieve positive event memories and negative event memories and to provide initial affect ratings and current affect ratings for each event. The results showed that increased levels of non-clinical depression were associated with reduced levels of the FAB. Indeed, at the highest level of depression, negative affect and positive affect faded equally.

Empirical evidence documents an excess of anxiety-mood disorders in the U.S. (Kessler, Petukhova, Sampson, Zaslavsky, & Wittchen, 2012), a relationship that extends into adolescence (Merikangas et al., 2010). Unlike their clinical counterparts, however, both subclinical depression and anxiety fall within normal boundaries of everyday human emotional experience (Fajkowska, 2013). The difference is that *subclinical* refers to a normal response combination of affect, cognitive interpretation, behavioral reaction, and desired outcome rather than the more disruptive clinical state (Wilt, Oehlberg, & Revelle, 2011).

From a social constructionist perspective, humans survive and even flourish because we have learned to make sense of each experience by generating culturally-appropriate personal meaning systems with which to interpret and if necessary act upon them (Yancu, 2011). Seen in this light, how people address everyday life is shaped by multiple complex factors rooted in both culture and context. Culture effectively standardizes the customs and rituals that formalize the human relationships according to the core groups’ value system. It, therefore, enables individuals and groups to reduce stress by anticipating events. Context, however, varies from moment to moment partly because of its dependency on available resources both intrinsic and extrinsic to the individual. When confronted by unanticipated events we typically respond by subjectively appraising their threat level. This response mechanism represents anxiety, an active relationship among stimulation or perceived threat, personality structure (Fajkowska, 2013), and cultural experience.

The research that we describe in the present article attempts to extend this individual-differences approach to the FAB into the domain of non-clinical trait anxiety. Several facts provide push toward such an extension. First, high trait anxiety is a very prevalent problem, even in its sub-clinical form (Fajkowska, 2013; Kessler et al., 2012; Wilt et al., 2011). Hence, research that links FAB to anxiety will apply to a large sub-population. Second, there is reason to expect that an FAB-anxiety relation will exist. For example, remember that dysphoria has already been explored in FAB research, and empirical evidence documents a robust association between depressive symptoms and anxiety (De Bolle, Decuyper, De Clercq, & De Fruyt, 2010; Fajkowska, 2013; Judah et al., 2013). Third, anxiety is seen by some as reflecting an emotion-regulation deficit associated with excessive worry (see Behar, DiMarco, Hekler, Mohlman, & Staples, 2009; Orgeta, 2011). It has been similarly argued that the FAB might reflect an individual’s ability to effectively engage in emotion regulation (Skowronski et al., 2014). The potential presence of such emotion-regulation deficits suggests that those who are highly anxious should show a disruption in the FAB that resembles the disruption pattern observed by Walker, Skowronski, Gibbons, et al. (2003) among dysphorics (i.e., no differential fading of affect in the highly anxious).

Clearly, such a finding should be important in its own right. However, such a finding would also help to shed light on a possible relationship between FAB and more extreme forms of anxiety, such as Post-Traumatic Stress Disorder (PTSD). In examining the etiology of PTSD, one of its unique features is that the PTSD diagnosis is predicated on having experienced a prior psychosocial stressor (Bonne, Grillon, Vythilingam, Neumeister, & Charney, 2004). Moreover, for those with PTSD, critical amounts of cognitive resources are invested to address the emotion-laden thoughts and feelings following the traumatic episode (Schweizer & Dalgleish, 2011). Indeed, for PTSD, affect does not seem to fade for very negative traumatic events. Given that difficulty in emotion regulation is a characteristic of PTSD (e.g., Ehring & Quack, 2010), demonstration that the FAB is moderated by anxiety might be seen as an initial step toward reconciling the FAB effect with the observation that in PTSD negative event-prompted affect does not fade.

## Study 1

Study 1 was conducted to determine whether individual differences in trait anxiety were related to how the positive affect and negative affect associated with autobiographical events were perceived to change from event occurrence to event recall. Previous research leads to the hypothesis that increased levels of anxiety should be associated with a reduced FAB, and that this reduced FAB should be a function of anxiety-related effects for both positive events and negative events.

### Material and method

#### Participants

A convenience sample of 98 African-American students between the ages of 18 and 49 years (66 females and 32 males) participated. All participants (Studies 1, 2 and 3) completed informed consent forms prior to participation, and received psychology course credit for their participation. The study design was in full compliance with the requirements of the Institutional Review Board of the university that served as the site where the research was conducted (i.e., the participants were treated in accordance with the *Ethical Principles of Psychologists and Code of Conduct*; American Psychological Association, 2002).

#### Anxiety assessment

Participants completed the Depression, Anxiety, and Stress Scale (DASS). The DASS is a 42-item survey that assesses non-clinical levels of depression, anxiety, and stress (Ng et al., 2007). For purposes of this study, only the anxiety subscale was used. Based on their scores on the anxiety subscale, participants were divided into three groups that were roughly equal in size: low anxiety (0-3; *n* = 35), moderate anxiety (4-9; *n* = 33), and high anxiety (10 and above; *n* = 30). The standard categories used when scoring the DASS are normal (0-7), mild anxiety (8-9), and moderate anxiety (10-14).

#### Recall procedure

Participants were given 20 min to recall 10 event memories. Five of these were to be negative, and five were to be positive. The order in which events of differing valence was recalled was randomly determined by a dice roll (41 participants recalled positive events first, 57 participants recalled negative events first). Participants were asked to describe each memory in 10 sentences and to include as many details as possible (e.g., time, location, sensory detail). A few participants were unable to recall all 10 events during the recall period, so across participants a total of 879 events were recalled.

#### Event affect ratings

After the time allotted for event recall had elapsed, participants were asked to rate initial affect and current affect for each of the 10 events. *Initial affect* referred to the affect associated with the event at occurrence. *Current affect* referred to the affect prompted by the event recollection. These ratings were both made on a 7-point scale ranging from -3 (*extremely unpleasant*) to +3 (*extremely pleasant*), with 0 being neutral. Both affect ratings were made in the same session. Changes in affect intensity were assessed for each item by calculating the difference between the ratings of initial affect and current affect.

#### Examination of event descriptions

After the completion of the study, two undergraduate research assistants examined the event descriptions. To get a measure of how important each event was to each participant’s sense of self, the research assistants counted the number of personal pronouns (e.g., *I*, *me*, *my*, *myself*) in each of the event descriptions.

### Data analyses

Before proceeding, three issues relevant to the data analyses need to be raised. The first issue concerns discrepancies between the event valence that was requested of participants and the valence of the events actually recorded. It was occasionally the case that when asked to record an event that produced a given emotional reaction at the event’s occurrence, participants sometimes recorded the event as producing no valence (a rating of 0) or gave the event a pleasantness rating that was the opposite of that requested. This happened 39 times, reflecting responses from 22 participants (responses from one subject for one item were missing). The inferential conclusions yielded by analyses did not change when these events were deleted from the dataset, so the analyses that we report below include these events.

A second issue concerns the use of difference scores in our analyses. We conceive of the experience of event-related affect as reflecting a range between extreme affect and none. However, the emotion associated with each event was measured on a bipolar scale ranging from +3 to -3. Hence, when an event changes valence from occurrence to recall, the difference score for the event (which could range from 4 to 6) will exceed the conceptual maximum (a change of three units). This occurred for 51 events spread across 32 participants. The analyses that we report below include these events. The inferential conclusions yielded by analyses did not change either when these events were deleted from the dataset or when the allowable amount of change was capped at three units.

The third issue that we raise concerns the fact that our analyses use a three-way categorical split on the anxiety individual difference predictor. Some authors have suggested that in analyses it is generally best practice to retain the continuous nature of individual differences measures such as the anxiety measure (e.g., MacCallum, Zhang, Preacher, & Rucker, 2002). However, DeCoster, Iselin, and Gallucci (2009) have argued and demonstrated that it is sometimes appropriate to use in analyses categorical variables that are derived from continuous measures. For example, in the clinical psychology domain, the anxiety categories that we used approximate the “standard” categories that are used for the anxiety measure that we employed, and this is one of the criteria that DeCoster et al. suggest can justify the use of such categories. Finally, the use of multiple categories for a predictor allows easy capture of quadratic relations between the predictor and an outcome variable, as well as easy description of interactions that might contain this quadratic component.

### Results and discussion

#### The FAB is moderated by trait anxiety: Analyses of change scores

The change scores were entered into a mixed-model stepwise regression analysis. The predictors in the model were the between-subjects variable of Participant Anxiety Level (low, medium, high), the within-subject variable of Requested Event Pleasantness (positive, negative), and the interaction between these two variables. The regression analysis was used instead of ANOVA to accommodate the fact that our data were not fully balanced: There were some missing data that derived from the facts that not all participants recalled 10 events, and that one subject did not provide ratings for all events. The regression approach allowed the data from these subjects to be retained in the analysis (ANOVA would not).

This regression analysis is conducted in much the same manner as the ANOVA approach, including separating variance into a between-subjects component and a within-subject component and testing the effects in those components against different error terms. In the present case, the only functional difference between the approaches is that in the regression approach, after accounting for between-subjects variance the main effect for event pleasantness is entered into the analysis in an initial step in which the results for that variable are interpreted. The interaction is then interpreted after entering it into the analysis in a second step. This stepwise procedure is used to account for the lack of independence between the event pleasantness variable and the Participant Anxiety Level × Event Pleasantness interaction.

**Figure 1.**
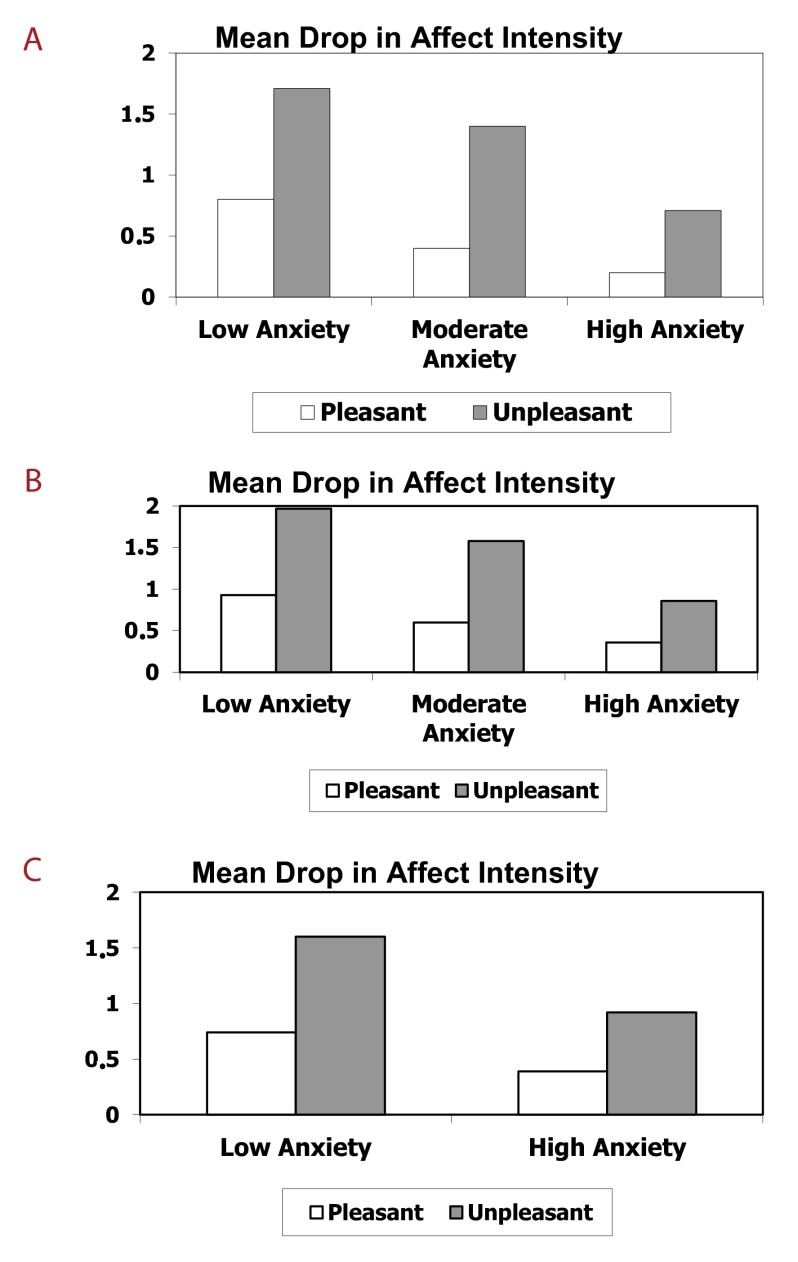
Change in affect as a function of event pleasantness and level of participant anxiety as assessed by the Depression, Anxiety, and Stress Scale (Experiment 1 [Panel A]) and the Beck Anxiety Inventory (Experiment 2 [Panel B] and Experiment 3 [Panel C]).

Indeed, the regression analysis yielded a Participant Anxiety Level × Event Pleasantness interaction, *F*(2, 878) = 3.68, η_p_^2^ = .008, *p* = .026.The means for this effect are depicted in Figure 1 (Panel A). As expected, the means show that the FAB was especially large for those in the low anxiety group. This bias was reduced but not eliminated for those in the moderate anxiety group and the high anxiety group. From these means, it is also obvious that across anxiety groups there is a meaningful valence effect. Negative events evinced more fading of affect (*M* = 1.45, *SD* = 0.43) from event occurrence to event recall than did positive events (*M* = 0.41, *SD* = 0.33), *F*(1, 880) = 155.77, η_p_^2^ = .15, *p* < .0001. However, there also appears to be a meaningful anxiety group effect, *F*(2, 95) = 3.57, η_p_^2^ = .024, *p* = .032. Across event valences, there appeared to have been more fading for people who were low in anxiety (*M* = 1.19, *SD* = 0.19) than for those who were moderate (*M* = 0.81, *SD* = 0.22) or high (*M* = 0.75, *SD* = 0.18) in anxiety. Subsidiary analyses confirmed that this anxiety group effect was significant for both positive events and negative events (*p*s = .0007, .035, respectively). A follow-up analysis was conducted to determine if anxiety was related to the initial event affect ratings in a way that would create distortion in the difference scores. The regression analysis of these initial event affect ratings did not yield a Participant Anxiety Level × Event Pleasantness interaction, *F*(2, 878) = 0.12, η_p_^2^ = .009, *p* = .885. An inspection of the means shows that the initial ratings for positive and negative events were similar for participants of all three anxiety levels (*M*_Negative_ = -2.65, -2.56, -2.66; *M*_Positive_ = 2.74, 2.63, 2.71).

#### Anxiety affects the content of event descriptions

The number of personal pronouns used in positive event descriptions and negative event descriptions was examined for participants across the three levels of anxiety. Positive events were described with slightly fewer personal pronouns than negative events (*M*_Positive_ = 1.2 words, *M*_Negative_ = 1.4 words), *F*(1, 96) = 0.33, *ns*. Low anxiety participants described their events with fewer personal pronouns than moderate and high anxiety participants (*M*_Low_ = 0.7 words, *M*_Moderate_ = 1.4 words, *M*_High_ = 1.7 words), *F*(2, 95) = 4.22, η_p_^2^ = .034. *p* = .02.

The results of Study 1 suggest that the effects of anxiety were twofold. First, increasing anxiety was associated with less affective fading for both positive event memories and negative event memories. Second, increasing levels of anxiety were associated with a FAB that was reduced, but not eliminated. We suggest that these results reflect the fact that for low anxiety participants, emotion regulation processes are able to help these individuals put the affect associated with event memories into perspective. For anxiety-prone participants, more anxiety disrupted these processes in such a way that served to retain the affective intensity of both positive events and negative events.

#### Potential weaknesses of Study 1

Study 1 had two potential weaknesses that may have affected the results. The first is that the study employed the DASS. This instrument has been shown to be a reliable measure of state anxiety, but it is limited to participants’ experiences in the most recent week. It would be useful to see if these results could be replicated using a measure that assessed prolonged state anxiety. Second, the recall procedure was a bit taxing in that a few participants had difficulty recalling 10 event memories in the allotted time, which resulted in a total of 39 missing events and the absence of the corresponding ratings for those events (17 positive events, 22 negative events, a total of 3.9% of the event data). A less-taxing procedure might yield a data set that is more complete than the data set obtained in Study 1.

## Study 2

Study 2 was conducted to again explore whether individual differences in trait anxiety were related to the extent to which the affect associated with autobiographical events was perceived to change from event occurrence to event recall. Study 2 differs from Study 1 in two important ways. First, to assess participant anxiety levels, Study 2 employed the Beck Anxiety Inventory (BAI; see Beck & Steer, 1993) rather than the DASS that was used in Study 1. Second, in an attempt to obtain a complete and balanced data set, participants were asked to retrieve only four rather than 10 memories. Despite these procedural differences, based on the results of Study 1 it was hypothesized that in Study 2 increased levels of anxiety should be associated with a reduced FAB, and that increased levels of anxiety would be associated with reduced levels of affective fading for both positive and negative events.

### Material and method

#### Participants

Fifty African-American students (38 females and 12 males) participated in this experiment. Participants were between the ages of 18 and 61 years.

#### Anxiety assessment

Participants completed the BAI. This score was used to place each participant into one of three groups: low anxiety (0-5; *n* = 17), mode-rate anxiety (6-13; *n* = 19), and high anxiety (14 and above; *n* = 14). For comparison, the standard categories used when scoring the BAI are normal (0-7), mild anxiety (8-15), and moderate anxiety (16-25). We deviated from these category cutoffs so that we could create groups of roughly equal size for our analyses.

#### Recall procedure

Participants were given 10 min to recall four event memories. Two of these were to be negative, and two were to be positive. The order in which events of a given valance was recalled was randomly determined by a dice roll for each participant (28 participants recalled positive events first, 22 participants recalled negative events first). Participants were asked to describe each memory in four to 10 sentences and to include as many details as possible (e.g., time, location, sensory detail). A total of 200 events were recalled.

#### Event affect ratings

After the time allotted for event recall had elapsed, participants were asked to rate initial affect and current affect for each of the four events. These ratings were both made on a 7-point scale ranging from -3 (*extremely unpleasant*) to +3 (*extremely pleasant*), with 0 being *neutral*. As in Study 1, both of these ratings were made in the same session. Changes in affect intensity were assessed for each item by calculating the difference between the ratings of initial affect and current affect.

#### Examination of event descriptions

After the completion of the study, two undergraduate research assistants examined the event descriptions. To obtain a measure of how detailed each description was, the research assistants counted the total number of words used to describe each event. To get a measure of how important each event was to each participant’s sense of self, the research assistants counted the number of personal pronouns in each of the event descriptions (e.g., *I*, *me*, *my*, *myself*).

### Results and discussion

The data for Study 2 were analyzed using the same analytic procedures and techniques that were employed in Experiment 1.

#### The FAB is moderated by trait anxiety: Analyses of change scores

The change scores were entered into a mixed-model regression analysis. The predictors in the model were the between-subject variable of Participant Anxiety Level (low, medium, high), the within-subject variable of Requested Event Pleasantness (positive, negative), and the interaction between these two variables.

The analysis yielded a Participant Anxiety Level × Requested Event Pleasantness interaction, *F*(2, 194) = 3.81, η_p_^2^ = .038, *p* = .029. The means for this effect are depicted in Figure 1 (Panel B). As expected, the means show that the FAB was especially large for those in the low anxiety group. This bias was reduced but not eliminated for those in the moderate anxiety group and the high anxiety group. From these means, it is also obvious that there is a meaningful FAB across groups: Negative events evinced more fading of affect (*M* = 1.47, *SD* = 0.16)from event occurrence to event recall than did positive events (*M* = 0.56, *SD* = 0.15), *F*(1, 194) = 90.45, η_p_^2^ = .32, *p* < .0001. The data also suggested that across event valences, there was more affect fading for people who were low in anxiety (*M* = 1.45, *SD* = 0.28) than for those who were moderate in anxiety (*M* = 1.09, *SD* = 0.18) or high (*M* = 0.61, *SD* = 0.22) in anxiety, *F*(2, 48) = 3.97, η_p_^2^ = .039, *p* = .021. A follow-up analysis was conducted to determine if anxiety had affected the initial event affect ratings in such a way that would create the distortion observed in the difference scores. The regression analysis did not yield a Participant Anxiety Level × Event Pleasantness interaction, *F*(2, 194) = 1.42, η_p_^2^ = .009, *p* = .575. Inspection of the means shows that the initial ratings for positive events and negative events were similar for participants at all three anxiety levels (*M*_Negative_ = -2.20, -2.29, -2.31; *M*_Positive_ = 2.58, 2.46, 2.59).

#### Anxiety is not related to the content of event descriptions

The number of personal pronouns used in positive event descriptions and negative event descriptions was examined for participants across the three levels of anxiety. Positive events were described with approximately the same number of personal pronouns as negative events (*M*_Positive_ = 1.1 words, *M*_Negative_ = 0.8 words), *F*(1, 194) = 1.18,*ns*. Likewise, there was no significant difference in the number of personal pronouns used by low, moderate, or high anxiety participants (*M*_Low_ = 0.7 words, *M*_Moderate_ = 1.1 words, *M*_High_ = 0.8 words), *F*(2, 48) = 1.09,η_p_^2^ = .004, *p* = .685.

#### Potential weaknesses of Studies 1 and 2

Studies 1 and 2 relied on retrospective recall of past events. Participants made ratings of initial affect and current affect in the same session. A critic might argue that changes in the affect prompted by events and the subsequent memories for such events can only be appropriately assessed by measuring event affect at the time an event occurred, not retrospectively. One reply to this criticism is that the extant research on the FAB has found that the FAB emerges from affect ratings regardless of whether those ratings are obtained using retrospective recall procedures or prospective diary procedures (see Skowronski et al., 2014; Walker & Skowronski, 2009) in which affect is recorded at the time of event occurrence and then the affect is assessed again at a later time when participants recall the event. However, a better response to the criticism would be to replicate the results of Studies 1 and 2 using these prospective diary procedures. Such procedures methodologically and temporally separate the ratings of initial affect and current affect. That is exactly what we did in Study 3.

## Study 3

Study 3 was conducted to determine whether individual differences in trait anxiety were related to how the affect associated with autobiographical events was perceived to change from event occurrence to event recall. Study 3 differed from Studies 1 and 2 in that it employed a diary procedure rather than a retrospective recall procedure. Nonetheless, the results obtained in Studies 1 and 2 lead to two primary hypotheses for Study 3: (a) Increased levels of anxiety should be associated with a reduced FAB, and (b) increased levels of anxiety should be associated with reduced levels of affective fading for both positive events and negative events.

### Material and method

#### Participants

Twenty-three African-American and two Caucasian students were recruited from the same university that was the source of the participants used in Studies 1 and 2. The participants included 17 females and eight males, and all participants were between the ages of 18 and 38 years.

#### Anxiety assessment

At the start of the study, participants completed the BAI. Each participant’s score was used to place the participant into one of two groups: low anxiety (0-11; *n* = 11) or high anxiety (13-40; *n* = 14). The present experiment used only two anxiety groups to create groups of comparable size in the smaller sample size. For comparison, the standard categories used when scoring the BAI are normal (0-7), and moderate anxiety (16-25).

#### Diary procedure

Participants were given five diary sheets and were instructed to keep a daily record of two unique events per day (one positive, one negative) for a period of 5 days. Thus, each participant was to provide 10 total event descriptions (five positive events, five negative events). Participants were instructed to record each event with descriptions of five to 15 sentences and to include enough details so that each event could be easily identified by them at a later time. Participants were asked to record the date of each event and to rate the event pleasantness on a scale from -3 (*extremely unpleasant*) to +3 (*extremely pleasant*) with 0 being *neutral*. The diary procedure produced 247 usable events: Three events had to be discarded because they were deemed to be too generic.

#### Memory testing

The event descriptions for each participant were transcribed into a spreadsheet program in a random order. After one week had passed from the date of the last event entered into the diary, in a testing session participants were presented with each of their event descriptions. For each event, participants were asked to read each event description and then make a series of ratings for each event before continuing on to the next event description. The testing procedure took approximately 30 min.

#### Event affect ratings

During the testing session, participants were asked to rate the current affect for each of their 10 events on a 7-point scale ranging from -3 (*extremely unpleasant*) to +3 (*extremely pleasant*), with 0 being *neutral*. Changes in affect intensity were assessed for each item by calculating the difference between the rating provided for each event as it was entered into the diary (event occurrence) and the current affect rating prompted by reading the event description (event recall).

#### Examination of event descriptions

After the completion of the study, two undergraduate research assistants examined the event descriptions. To get a measure of how detailed each description was, the research assistants counted the total number of words used to describe each event. To get a measure of how important each event was to each participant’s sense of self, the research assistants counted the number of personal pronouns (e.g., *I*, *me*, *my*, *myself*) in each of the event descriptions.

### Results and discussion

#### The FAB is moderated by trait anxiety: Analyses of change scores

The change scores were entered into a mixed-model regression analysis. The predictors in the model were the between-subjects variable of Participant Anxiety Level (low, medium, high), the within-subject variable of Requested Event Pleasantness (positive, negative), and the interaction between these two variables.

The analysis yielded a Participant Anxiety Level × Requested Event Pleasantness interaction, *F*(1, 243) = 7.49, η_p_^2^ = .04, *p* = .007. The means for this effect are depicted in Figure 1 (Panel C). As expected,they show that the FAB was especially large for those in the low anxiety group and that this bias was reduced but not eliminated for high anxiety participants. From these means it is obvious that there is a meaningful FAB across groups: Negative events evinced more fading of affect (*M* = 1.26, *SD* = 0.01) from event occurrence to event recall than did positive events (*M* = 0.58, *SD* = 0.13), *F*(1, 243) = 104.12, η_p_^2^ = .31, *p* < .0001. Although as in prior studies, collapsing across event valence, high anxiety participants evinced less fading of affect than low anxiety participants, the anxiety group main effect was not statistically significant in Study 3, *F*(1, 12) = 1.19, η_p_^2^ = .005, *p* = .27. A follow-up analysis was conducted to determine if anxiety was related to the initial event affect ratings in such a way that those initial ratings would distort the difference scores. The regression analysis did not yield a Participant Anxiety Level × Event Pleasantness interaction, *F*(1, 243) = 1.70, η_p_^2^ = .021, *p* = .775. An inspection of the means shows that the initial ratings for positive and negative events were similar for participants of both anxiety levels (*M*_Negative_ = -2.20, 2.37; *M*_Positive_ = 2.56, 2.58).

#### Anxiety is related to the content of event descriptions

The number of personal pronouns used in positive and negative event descriptions was examined for low anxiety participants and high anxiety participants. Positive events were described with slightly fewer personal pronouns than negative events (*M*_Positive_ = 1.8 words, *M*_Negative_ = 1.6), *F*(1, 243) = 1.15, η_p_^2^ = .005,*ns*. Low anxiety participants described their events with fewer personal pronouns than high anxiety participants (*M*_Low_ = 1.4 words, *M*_High_ = 1.9 words), *F*(1, 12) = 3.51, η_p_^2^ = .042. *p* < .024.

## General Discussion

### Replication of the FAB and implications for anxiety

The results of the studies described in this article serve to replicate and extend the findings obtained in previous research on the FAB. The FAB robustly emerged in all three of the studies that we report in the present article. Moreover, in all three of our studies, the FAB was especially robust in those subjects who were low in anxiety, and was weakened but not eliminated for those who were high in anxiety. Moreover, the results of all three studies indicated that the FAB is disrupted by trait anxiety in a very particular way: Higher anxiety was associated with less affective fading for both positive and negative events. This last result suggests that increased levels of anxiety may produce a heightened sense of arousal that serves to amplify the perceived emotional qualities of remembered events. The net result seems to be that the emotions of the past seem to fade less for anxiety prone individuals than for their “normal” counterparts, regardless of whether the emotion is negative or positive.

The relation between anxiety and affective fading observed in this study may be related to *rumination*, an emotion regulation strategy often employed by individuals suffering from anxiety. The repeated recollection of event memories could serve to amplify the emotional content of those memories, thus reducing fading affect. One way to test this possibility is to examine the event descriptions provided by participants. If people higher in anxiety have been ruminating over their memories, it is reasonable to suspect that their memories might be described with more references to the self than the memories of less anxious individuals. That is exactly what happened. For participants in Studies 1 and 3, higher levels of anxiety were associated with memories with more references to the self. This effect of anxiety did not interact with event valence.

The present findings also converge with results provided by Mennin, Heimberg, Turk, and Fresco (2005) who, using the Berkely Expressivity Questionnaire (BEQ) to assess differences in emotion, found that individuals suffering from a generalized anxiety disorder often report heightened emotional experiences and a generally poorer understanding of their emotions. This is hardly surprising. Trait anxiety is the manifestation of a negative emotional state evoked by the anticipated presence of a real or perceived threat, and it will gene-rate very real consequences in the individual’s physiological response (Sorrells, Caso, Munhoz, & Sapolsky, 2009). As Gardner (2008) notes, in the post-9/11 world many people continue to feel uneasy at the sound of low flying planes. In other words, the memory of that event evokes a mild form of anxiety rooted in a sense of uncertainty, even though no imminent threat is present.

Although some degree of anxiety is adaptive in that it fosters a healthy sense of caution about potential threats, too much anxiety can be debilitating, disrupting one’s ability to thrive and resulting in an anxiety disorder (Gardner, 2008). In this regard, it should be noted that the focus of the three studies that we report in this article was individuals who were identified as having subclinical levels of trait anxiety. As distinct from its clinical counterpart, trait anxiety is directly related to negative affect (Olatunji & Wolitzky-Taylor, 2009) and refers to those who are more likely to respond fearfully to various stressors. For example, in the three studies that we report, those who scored as moderate or high in trait anxiety were less likely to show a FAB for either positive memories or negative memories in contrast to those who expressed low or no anxiety. This may be because high-trait anxious individuals are more likely to rely on a threat-related, interpretive bias (Bishop, 2007) when evaluating an event memory. Alternatively, it may be that low-anxiety individuals are more prone to appraise the memory of a negative event as a challenge to be overcome or addressed (Benight, 2011).

More importantly, other studies have shown that individuals oriented towards higher levels of anxiety need not even be aware at a conscious level of the threat-related distractions competing for their attention (Bishop, 2007). Here, higher levels of anxiety seem to be associated with a general heightened experience of affect, which likely affects how individuals experience the world and how they recall autobiographical memories. These data suggest that, in effect, high anxiety group members may be relatively unsuccessful at engaging healthy coping responses.

This last point brings up the issue of causality. It is possible that differences in the FAB produced subtle cognitive vulnerabilities in individuals, who then later became susceptible to trait anxiety. This possibility seems unlikely because such an assertion stands in direct contrast with previous findings. Our research has shown the FAB to be a stable phenomenon operating in autobiographical memory, but a phenomenon that can vary according to both circumstances and individual difference variables. We have found that the FAB is causally influenced by social rehearsal: The more a person talks about a negative event, the greater the emotion fades, while greater social rehearsal leads to less emotional fading for positive events (Skowronski et al., 2004). We have also found that dysphoria can disrupt the FAB, with individuals expressing higher levels of dysphoria showing a substantially weakened FAB (Walker, Skowronski, Gibbons, et al., 2003). Given such findings, it is more likely that the FAB serves as an indicator of the overall health of an individual’s autobiographical memory. When a person is emotionally stable, routinely engages in social discourse, and allows enough time to pass, the FAB should be present, helping to maintain a generally positive worldview.

Ultimately, the relationship between relatively high levels of trait anxiety and a heightened degree of affect sensitivity could shed light on the etiology of other anxiety-related disorders (e.g., social anxiety, panic disorders). For example, individuals with PTSD are unable to discriminate between threatening and non-threatening stimuli (Bonne et al., 2004). They expend large amounts of internal resources to live with the thoughts and feelings that accompany PTSD, taxing their ability to function effectively (Schweizer & Dalgleish, 2011). Yet, many people who experience a traumatic event do not develop PTSD. One may speculate that this may be because they have a variety of effective coping strategies to draw on to alleviate distress. It could be that in a psychologically healthy individual, the emotional memory of the event or experience is dampened over time so that internal resources may be used elsewhere to preserve well-being. While more research is needed to examine the relations between specific kinds of anxiety (e.g., post-traumatic stress) on the FAB, in the case of trait anxiety, heightened arousal seems to disrupt the affective fading for both positive and negative autobiographical events.

One issue of note is that the data that we collected mostly were obtained from African-American students. While this may limit the generalizability of the present results, there are at least two reasons to suspect that this concern should not be overstated. First, the FAB seems to be a pan-cultural phenomenon: It has been found to be present in a number of cultures around the world, including samples in Africa, Europe, and North America (Ritchie et al., in press). Second, the findings of the present study conceptually replicate previous research on the impact of anxiety on emotion regulation, which has found that individuals suffering from anxiety have difficulty minimizing the emotions associated with life experiences (Mennin et al., 2005).

The studies that have been conducted in the last 20 years have shown the FAB to be a robust finding that holds considerable theoretical interest. Moreover, the relationship of the FAB to trait anxiety and dysphoria means that FAB may have some degree of diagnostic potential for both the presence of and the emergence of emotional disorders.
